# Correction: Segregation of Ca2^+^ signaling in olfactory signal transduction

**DOI:** 10.1085/jgp.20221316506062023c

**Published:** 2023-06-09

**Authors:** Hiroko Takeuchi, Takashi Kurahashi

Vol. 155, No. 4 | https://doi.org/10.1085/jgp.202213165 | February 14, 2023

**Correction: Sniffing out Ca2^+^ signaling in olfactory cilia**

Ben Short

Vol. 155, No. 4 | https://doi.org/10.1085/jgp.202313378 | March 22, 2023

Takeuchi and Kurahashi determined that the data presented in Fig. 6, D and G, is better presented as original data values in order to allow an accurate assessment of the experimental situation. Additionally, the label “Normalized” was added to panels E and H to clarify that the data presented is superimposed on the current waveform. These corrections do not affect the conclusions of this paper. The corrected version of the figure appears here.

**Figure 6.**
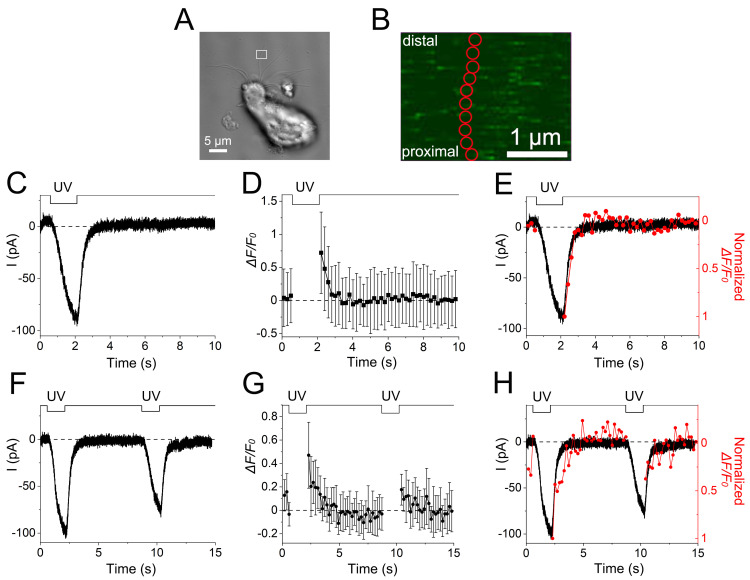


The Research News commentary about this article used the image from Fig. 6 H. The image in this commentary has also been updated.

The errors appear in PDFs downloaded before June 7, 2023.

